# MAFcounter: an efficient tool for counting the occurrences of k-mers in MAF files

**DOI:** 10.1186/s12859-025-06172-7

**Published:** 2025-05-30

**Authors:** Michail Patsakis, Kimonas Provatas, Aris Karatzikos, Charalampos Koilakos, Ioannis Mouratidis, Ilias Georgakopoulos-Soares

**Affiliations:** 1 Department of Biochemistry and Molecular Biology, Institute for Personalized Medicine, The Pennsylvania State University College of Medicine, Hershey, PA USA; 2 Huck Institute of the Life Sciences, Pennsylvania State University, University Park, PA USA

**Keywords:** k-mer counting, Genomics, Proteomics, Multiple sequence alignment

## Abstract

**Motivation:**

With the rapid expansion of large-scale biological datasets, DNA and protein sequence alignments have become essential for comparative genomics and proteomics. These alignments facilitate the exploration of sequence similarity patterns, providing valuable insights into sequence conservation, evolutionary relationships and for functional analyses. Typically, sequence alignments are stored in formats such as the Multiple Alignment Format (MAF). Counting k-mer occurrences is a crucial task in many computational biology applications, but currently, there is no algorithm designed for k-mer counting in alignment files.

**Results:**

We have developed MAFcounter, the first k-mer counter dedicated to alignment files. MAFcounter is multithreaded, fast, and memory efficient, enabling k-mer counting in DNA and protein sequence alignment files with a wide variety of features for k-mer analysis.

**Availability:**

MAFcounter is released under GPL license as a suite of binary C++ applications and is available at: https://github.com/Georgakopoulos-Soares-lab/MAFcounter.

**Supplementary Information:**

The online version contains supplementary material available at 10.1186/s12859-025-06172-7.

## Introduction

The rapid progress in high-throughput sequencing technologies has enabled the generation of reference genome and proteome assemblies across organisms from all domains of life and of different individuals across human populations in an ever-increasing pace [1–4]. Genomes and proteomes are composed of nucleotide or peptide sequences, respectively, which can be divided into fixed-length substrings called k-mers, where *k* represents the specified length [5]. The frequency of k-mers varies substantially across genomes and proteomes of different species as well as in genomic sub-compartments [6–9].

Counting the occurrence of all k-mers in biological sequences is a crucial step in many bioinformatic applications such as genome assembly, sequence comparison, sequence clustering, error correction of sequencing reads, and genome size estimation [10–14]. K-mers hold significant potential for understanding biological processes, as their occurrences can reveal key aspects of genomic features, including functional elements, repetitive sequences, transcription factor binding sites and variations in the genome, as well as reflect the imprints of DNA damage and repair patterns [15–18]. Proteomes, along with genomes, also play a critical role in k-mer counting analysis by providing insights into coding sequences, functional annotations, structural variations, and evolutionary processes (Bourgeas et al. 2023). Multiple k-mer counting tools have been developed including Jellyfish [19], DSK [20], Gebril [21], Meryl [22], KMC3 [23] and KCOSS [24] among others, all of which work on a single set of sequences, most often provided in FASTA file format. Nevertheless, there is no method that can count k-mer occurrences in multiple alignment files.

Here, we developed MAFcounter, the first k-mer counter tool for multiple alignment files. MAFcounter takes as input a MAF file and calculates the occurrences of k-mers across each sequence in the alignment, handling both genomic and proteomic sequences effectively within multiple alignment contexts.

## Materials and methods

### MAF file format definition

The Multiple Alignment Format (MAF) is a widely used file format designed to store multiple sequence alignments, particularly at the genomic level. Unlike formats optimized for single-protein or short DNA region alignments, MAF accommodates whole-genome alignments while handling complexities such as forward and reverse strand orientations and fragmented alignments across multiple genomic regions. MAF files are structured as a series of alignment blocks, each beginning with an “a” line, which may contain a score representing the alignment quality. Within each block, sequences are listed on “s” lines, where each sequence is associated with a specific genome, its start position, length, strand orientation, total genome size, and the aligned sequence itself. The first sequence in each alignment block serves as the reference genome to which all other sequences are aligned. In addition to “a” and “s” lines, MAF files can include optional lines such as “e”, “q”, and “i”, which provide information about gaps, sequence quality, and contextual details, though they are not required for a valid MAF file. Further information about the MAF format is provided by the UCSC Genome Browser (https://genome.ucsc.edu/FAQ/FAQformat.html#format5*)* [25].

### Data used for performance comparison

For performance comparisons, we used the compressed alignment file from the Human Pangenome Project (HPP) [25]. After decompressing the file, we extracted different portions of it for benchmarking purposes. To evaluate our tool in comparison with other k-mer counting tools, we selected two benchmark sizes: a small benchmark, consisting of 1/100th of the original file (resulting in a 2GB uncompressed MAF file), and a large benchmark, consisting of 1/10th of the original file (resulting in a 26.5GB uncompressed MAF file). The original alignment file is available at https://github.com/human-pangenomics/hpp_pangenome_resources, and the benchmark datasets used in our study can be accessed via Zenodo at: https://zenodo.org/records/14927620.

### Implementation of mafcounter

MAFcounter is a multi-threaded command-line tool implemented as a set of C++ programs. It utilizes the Google SparseHash library to optimize memory usage in hash-based data structures for small k-mer lengths and the Boost Multiprecision cpp_int library for larger k-mers. The implementation supports various command-line options, including different output formats and filtering capabilities tailored for MAF files (Supplementary Fig. 1). It provides two types of output. The default option generates a map-reduced output that consolidates all k-mers into a single file, where each k-mer is followed by the genome IDs in which it was found. Alternatively, a separate output file for each genome ID, where each file contains the k-mers specific to that genome, is also possible. MAFcounter enables k-mer counting for k up to 64 in the case of DNA/RNA alignments and for protein alignments up to 25.

MAFcounter accepts both uncompressed and compressed MAF files as input. If a gzipped MAF file is detected, the program automatically extracts the .gz file into the current directory before proceeding with the main algorithm, allowing MAFcounter to seamlessly handle compressed files. Once the extraction is complete, the tool partitions the MAF file into N chunks, assigning each chunk to a separate thread to distribute the computational workload. Once chunks are defined, a k-mer counting strategy is selected based on the k-mer length (k). For k ≤ 10, a hashmap-based approach using Google Dense HashMap is employed. For k > 10, a disk-based approach is used to optimize memory consumption, following strategies commonly implemented in existing k-mer counting tools.

For k ≤ 10, the algorithm assigns chunks to threads in a round-robin manner. Each thread performs a sliding window operation on the ungapped ‘s’ lines of its assigned MAF chunk, storing encoded k-mers and their frequencies. This representation allows encoding of up to 4^10^ k-mers while associating each k-mer with its respective genome ID. The thread updates a local hashmap with k-mers as keys and genome ID - count pairs as value. After all threads complete their tasks, the main thread sequentially merges individual hashmaps to prevent excessive memory usage. The final aggregated hashmap is serialized and stored on disk. This method is sufficient for smaller k-mer sizes without significantly impacting performance or memory efficiency.

For k > 10, MAFcounter implements a disk-based counting approach, inspired by the first version of KMC [26], that incorporates prefix-based grouping, external radix sorting, and parallelized disk writing to maximize I/O performance. The algorithm initializes N reader threads and M package manager threads. Each reader thread performs a sliding window operation on ungapped ‘s’ lines, encodes k-mers with a 2-bit representation, and retrieves the corresponding genome ID. The encoded k-mers are then assigned to bins based on the first 10 bits (or the first 5 nucleotides, denoted as Prefix). When a bin surpasses a predefined threshold, it is partially sorted based on the next 10 bits (or the following 5 nucleotides, termed the Infix). The Prefix and Infix are then removed, leaving a suffix k-mer, which contains the remainder of the k-mer and the genome ID. Genome IDs are consistently stored in the 8 least significant bits, with the suffix k-mer encoded in the remaining bits. After sorting and compaction, the completed bin is placed into a queue monitored by the package manager threads. These threads manage packages for each Prefix, maintaining the same format as partially sorted bins. Packages are merged efficiently based on Infix values while preserving order. Once a package reaches a predefined element threshold, it is written to disk. This process continues until all sequences in the MAF file are processed, and all packages are stored. Following the completion of package management, the main thread launches N + M sorter threads, which process disk-stored packages in a round-robin manner based on Prefix. The sorter threads load, perform a full radix sort, and write in parallel to the binary output file after having calculated byte offsets of each package.A metadata file containing byte offsets for each Prefix is also generated to enable efficient random access.

The binary outputs of MAFcounter are compatible with maf_counter_tools and maf_counter_dump, facilitating downstream analyses. The hybrid counting strategy employed by MAFcounter enables competitive execution times while maintaining bound memory usage, making it suitable for large-scale k-mer analysis in large multiple sequence alignments.

### MAF file specific features

We also implemented a series of MAF specific features for the analysis of k-mer outputs. The primary output is a single-file format generated by maf_counter_dump. This file has a single line per k-mer and in that line space separated genomeID: count pairs. This file can be used to perform differential analysis on genome IDs, identify k-mers that are differentially present between the genome IDs and more. Another MAF specific feature is using the parameter –genome_ids so that the user can keep sequences only for that specific genome IDs, this eliminates the need for pre-processing or post-processing on genomic data and makes MAFcounter more flexible as a k-mer counter. Another feature that falls into the MAF specific category is the option to perform k-mer counting on base pairs that have a quality score between a specific range using the –min_q_level and –max_q_level. When MAF files have ‘q’ lines these are used to associate each base pair from the above ‘s’ line with a quality score from 1–9 and F (complete). Using this feature the user can perform k-mer analysis on high or low confidence regions. A feature that follows the same principle but is applied at the block level is the ability to perform k-mer counting on blocks that have an alignment score between a specific range using the –min_a_score and –max_a_score. Alignment scores are derived from alignment software and are used as a metric to estimate how well the sequences align in the block. Using this feature the user can flexibly perform k-mer analysis on blocks that align less or more.

The maf_counter_tools utility is designed for advanced analysis of k-mer datasets derived from multiple sequence alignments. It facilitates the identification of k-mers with high variance across genomes by computing the standard deviation of k-mer counts. This functionality is implemented using a multi-threaded algorithm based on min-heaps. Additionally, maf_counter_tools provides statistical summaries of k-mer distributions per genome ID, including minimum, maximum, mean, median, variance, and skewness, computed efficiently through a concurrent algorithm that processes data in chunks. The utility also enables filtering of k-mers from the binary database based on logical expressions applied to genome-specific counts, allowing users to define conditions involving comparison operators (<, >, =) and logical operators (&&, ||), with support for parentheses to modify precedence. Furthermore, maf_counter_tools allows querying of k-mers using a comma-separated list, an input file, or regular expressions, enabling applications such as the detection of transcription factor binding sites or G-quadruplexes following the consensus d(G_3+_N_1 − 7_G_3+_N_1−7_G_3+_N_1−7_G_3+_) motif [27].

### Performance comparison against other k-mer counting software

Because MAFcounter relies on extensive temporary-file I/O during its disk-based counting stages, its overall speed and memory profile are strongly tied to disk throughput. For optimal results, use of an SSD or equally fast storage device is recommended.

**Fig. 1:**
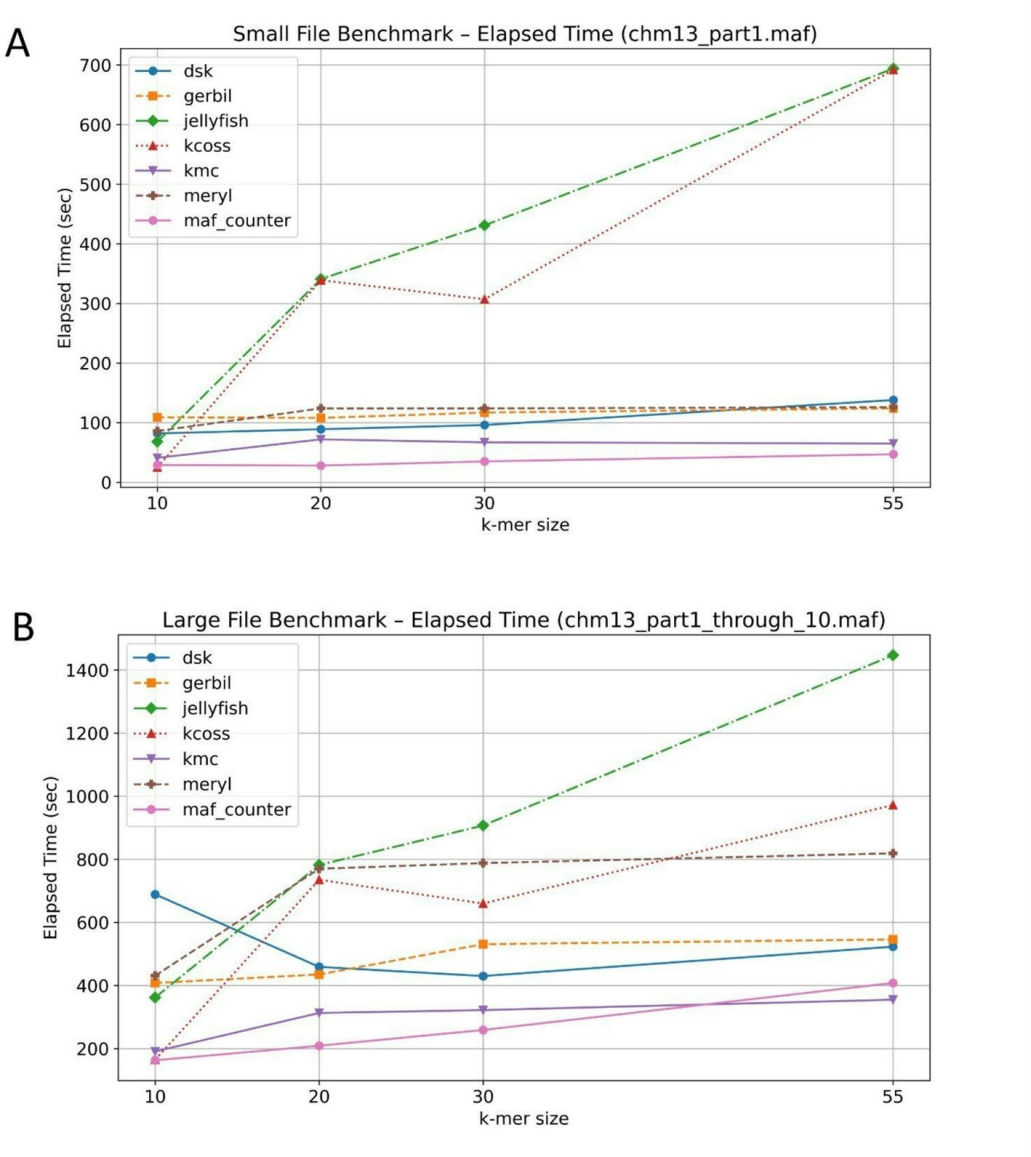
Performance comparison of MAFcounter with k-mer counting tools. **A** Execution time over K, for K = 10,20,30,55 in a small MAF file. **B** Execution time over K, for K = 10,20,30,55 in a large MAF file

To test the performance of MAFcounter against other k-mer counting software we created a pipeline that uses the workload manager Slurm was used to submit k-mer counting jobs that are fine-tuned for each k-mer tool to utilize the most out of 64GB of RAM and 24 cores of Intel(R) Xeon(R) Gold 6342 CPU @ 2.80 GHz.The disk that all benchmarks were conducted on is an Intel Optane SSD DC P5800X Series. We tested MAFcounter against Jellyfish, KMC3, KCOSS, DSK, Meryl and Gerbil, some of the most commonly used k-mer counters. Benchmarks are designed using an orchestrator bash script for each tool that submits k-mer counting jobs after the MAF file is broken into FASTA as k-mer counters do not work directly on MAF files. The breaking of MAF file to FASTA has a very low constant time overhead but it is a necessary step and as it will be evident in the comparison section MAFcounter is faster even after accounting for a small overhead to convert MAF files to FASTA for other tools. The constant overhead to convert the small file (chm13_part1.maf) to fasta is 17 s and the overhead to convert the large file (chm13_part1_through_10.maf) is 137 s whereas the memory overhead is essentially zero for both files. All tools were fine-tuned to utilize 24 threads and whenever there was an option to use more memory we enabled that for the tool tested, such as the -r turn on RAM-only mode option for KMC3 or memory = 64 option for Meryl. The same principles have been followed in the fine-tuning of Jellyfish, Gerbil and KCOSS and DSK. We employed this methodology to ensure the competing tools were configured with their fine-tuned settings against MAFcounter while we also run the benchmarks with the default parameters. The execution time of MAFcounter compared to the other tools, picking the fastest time among the default and fine-tuned parameters for other tools is shown at the two graphs below (Fig. 1). The time memory and disk results for the small file (chm13_part1.maf) and the large file (chm13_part1_through_10.maf) broken into the default and fine-tuned parameters are shown in four different tables grouped by K = 10,20,30,55 (Supplementary Table 1). An analysis of the graph for both files indicates that MAFcounter consistently demonstrates the highest processing speed among the evaluated tools, with the exception of a slight performance advantage observed for KMC at k = 55. An examination of the performance table reveals that MAFcounter exhibits higher memory consumption and disk usage compared to the other tools. This difference can be attributed to several factors. First, alternative tools in the existing pipeline perform N k-mer counting operations—where N corresponds to the number of FASTA files representing individual genomes in the MAF, each utilizing multi-threading. This results in faster processing per FASTA file and reduced memory usage, as each tool handles smaller input sizes rather than the entire MAF file at once. Second, MAFcounter uses a prefix-based method to support features specific to MAF files. This method requires each k-mer to be encoded with its genome ID, which is necessary for correctly generating a single, combined output file and the downstream program maf_counter_tools to scale efficiently. Other k-mer counting tools do not natively support this type of output. To produce a similar result using those tools, one would need to use a post-processing script based on a hash map. However, this approach becomes inefficient for larger k-mer sizes (e.g., k > 10), as it is both memory-intensive and slow while also not trivial to implement.

### Multithreading and scaling

**Fig. 2:**
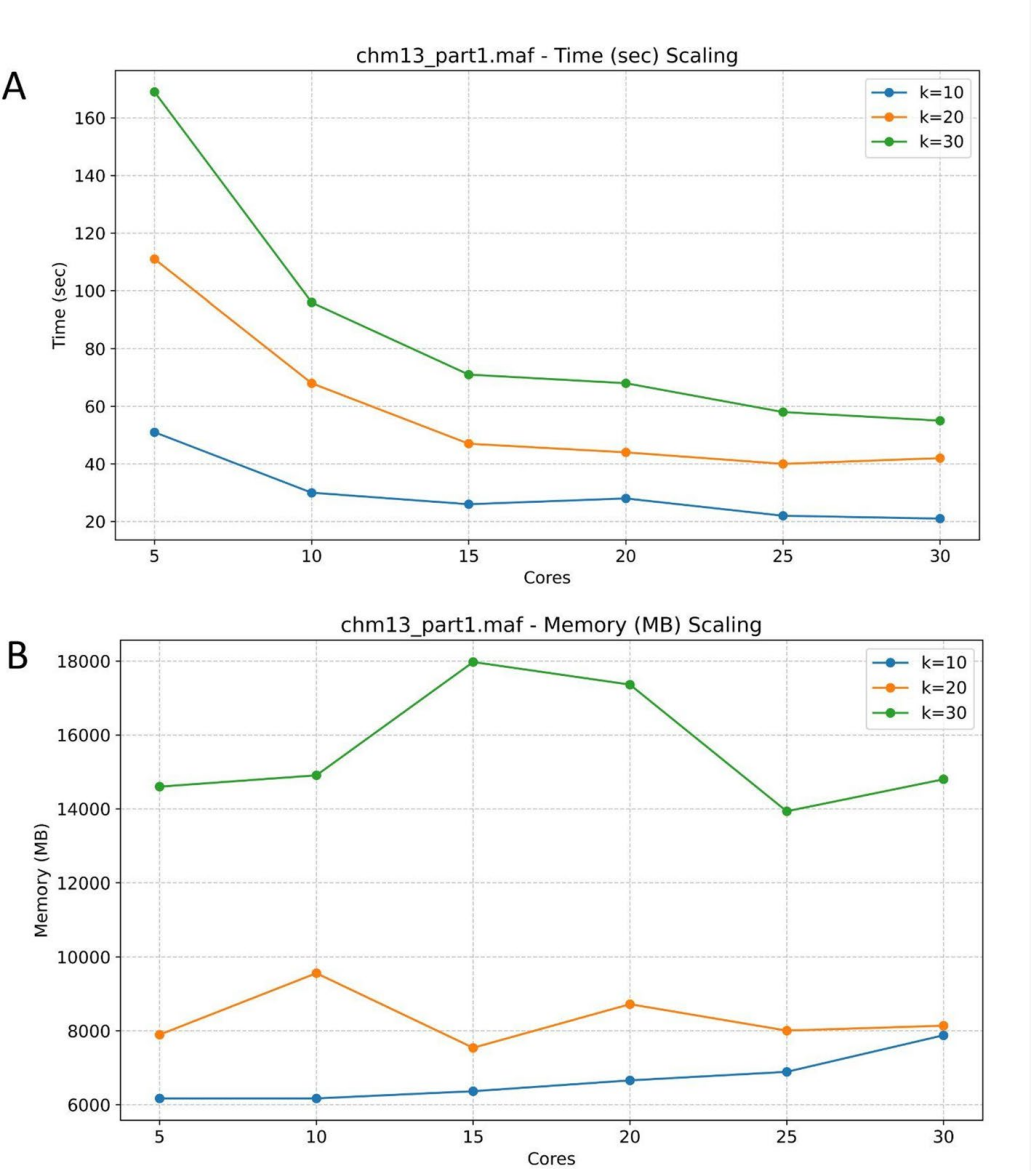
Computational Performance Scaling with Core Usage. **A** Memory time shown as a function of cores used. **B** Time in seconds shown as a function of the number of cores used. Results shown for k-mer lengths of 10 bp, 20 bp and 30bps

MAFcounter employs multithreading whenever concurrent processing can be applied. MAFcounter uses either N reader threads and M package manager threads or a total number of threads that is then split by the algorithm into the N and M values above. When K is less or equal than 10 a hashmap-based approach is employed using N readers that construct a local hashmap that is then merged into the final HashMap when readers complete the parsing. When K is more than 10 a disk based approach is used and multiple readers read the file while package managers take the full bins from readers and concurrently merge them into packages and write them to disk if they reach a specific threshold. When these package managers complete another set of sorters load the flushed packages into memory, sort them fully using radix sort and dump them in the final output file. In general the bin threshold, the package manager threshold and the number of sorted bins affect memory usage. We decided to have constant values for both thresholds that experimentally yield bound memory usage and to let readers and package managers vary based on user preference. For configurations that do not specify separate reader and package-manager thread counts, a single total thread count may be apportioned automatically into 30% readers and 70% package-manager threads, which experimental results show minimizes the imbalance in their completion times. In general, systems with higher I/O throughput and greater available memory benefit from a larger proportion of reader threads, whereas environments with lower I/O performance and more constrained memory should reduce the number of reader threads to avoid excessive memory consumption. To monitor MAFcounter’s memory usage as threads increase we created a pipeline for K = 10,20,30 and thread count 5,10,15,20,25,30. We observed that MAFcounter scales very well in terms of time for various values of K whereas memory consumption stays within reasonable limits even for larger files. (Fig. 2).

To evaluate the performance of MAFcounter on protein MAF files, we conducted a series of scalability tests focused on execution time and memory usage as a function of the number of threads. Using a 7.6 MB input file, we varied the thread count from 2 to 8 in steps of 2 and evaluated k-mer sizes ranging from 5 to 25 in increments of 5, thus covering a representative range of peptide k-mer lengths. The results indicate that MAFcounter scales efficiently with increasing thread count (Supplementary Fig. 2). While memory usage was observed to be higher compared to the DNA counterpart, this is consistent with expectations, given that the amino acid alphabet (up to 25 symbols, when accounting for ambiguity codes and special characters) is significantly larger than the nucleotide alphabet (4 symbols). Although a theoretical analysis suggests a sixfold increase in memory usage due to the constraints of binary encoding across 32, 64, and 128-bit variables, in practice, we followed a hashmap-based approach due to the scarcity and smaller sizes of protein alignments, which results in substantial but manageable memory requirements.

To evaluate the performance of the query command we benchmarked the query tool on a 370 GB binary database comprising all 55-mers extracted from the “part1_through_10.maf” file (25.7 GB) (Supplementary Fig. 3). The workload consisted of 100 query k-mers evenly distributed among all p1 prefixes. To assess parallel performance, we varied the thread count starting at 1 and 2, then in steps of 2 up to 16. The tool exhibited excellent strong scaling, with execution time decreasing steadily to a minimum of 6.32 s at 10 threads; beyond this point no further improvements were observed.

## Discussion

In this study, we developed MAF Counter, a robust counter tool designed to efficiently analyze sequence alignments and quantify the occurrence of k-mers. MAF Counter provides a valuable resource for researchers working on comparative genomics, evolutionary biology, and other fields where sequence alignment is critical and counting k-mer occurrences in a first step in data interpretation. By enabling rapid and accurate counting of k-mers in multiple alignments, MAFcounter simplifies large-scale genomic analyses and reduces the computational burden associated with handling extensive datasets. The program’s modularity and scalability allow for its integration into existing pipelines, enhancing its applicability to a wide range of research projects, from basic biology to clinical genomics.

## Electronic supplementary material

Below is the link to the electronic supplementary material.

Supplementary Material 1.

## Data Availability

No datasets were generated or analysed during the current study.

## Code availability

The source code for MAFcounter is provided at: https://github.com/Georgakopoulos-Soares-lab/MAFcounter. The source code for the benchmarks is provided at: https://github.com/Georgakopoulos-Soares-lab/MAFcounter-pipelines. Files needed to reproduce the benchmarks are found at: https://zenodo.org/records/15304631.

## References

[1] Rhie A, McCarthy SA, Fedrigo O, Damas J, Formenti G, Koren S, et al. Towards complete and error-free genome assemblies of all vertebrate species. Nature. 2021;592:737–46. 33911273 10.1038/s41586-021-03451-0 PMC8081667

[2] Darwin Tree of Life Project Consortium. Sequence locally, think globally: the Darwin tree of life project. Proc Natl Acad Sci USA. 2022;119. 10.1073/pnas.2115642118 PMC8797607 35042805

[3] Karczewski KJ, Francioli LC, Tiao G, Cummings BB, Alföldi J, Wang Q, et al. The mutational constraint spectrum quantified from variation in 141,456 humans. Nature. 2020;581:434–43. 32461654 10.1038/s41586-020-2308-7 PMC7334197

[4] Exposito-Alonso M, Drost H-G, Burbano HA, Weigel D. The Earth biogenome project: opportunities and challenges for plant genomics and conservation. Plant J. 2020;102:222–9. 31788877 10.1111/tpj.14631

[5] Moeckel C, Mareboina M, Konnaris MA, Chan CSY, Mouratidis I, Montgomery A, et al. A survey of k-mer methods and applications in bioinformatics. Comput Struct Biotechnol J. 2024;23:2289–303. 38840832 10.1016/j.csbj.2024.05.025 PMC11152613

[6] Georgakopoulos-Soares I, Yizhar-Barnea O, Mouratidis I, Hemberg M, Ahituv N. Absent from DNA and protein: genomic characterization of nullomers and nullpeptides across functional categories and evolution. Genome Biol. 2021;22:245. 34433494 10.1186/s13059-021-02459-z PMC8386077

[7] Chor B, Horn D, Goldman N, Levy Y, Massingham T. Genomic DNA k-mer spectra: models and modalities. Genome Biol. 2009;10:R108. 19814784 10.1186/gb-2009-10-10-r108 PMC2784323

[8] Chantzi N, Mareboina M, Konnaris MA, Montgomery A, Patsakis M, Mouratidis I, et al. The determinants of the rarity of nucleic and peptide short sequences in nature. NAR Genom Bioinform. 2024;6:lqae029. 38584871 10.1093/nargab/lqae029 PMC10993293

[9] Koulouras G, Frith MC. Significant non-existence of sequences in genomes and proteomes. Nucleic Acids Res. 2021;49:3139–55. 33693858 10.1093/nar/gkab139 PMC8034619

[10] Kelley DR, Schatz MC, Salzberg SL. Quake: quality-aware detection and correction of sequencing errors. Genome Biol. 2010;11:R116. 21114842 10.1186/gb-2010-11-11-r116 PMC3156955

[11] Allam A, Kalnis P, Solovyev V. Karect: accurate correction of substitution, insertion and deletion errors for next-generation sequencing data. Bioinformatics. 2015;31:3421–8. 26177965 10.1093/bioinformatics/btv415

[12] Salmela L, Schröder J. Correcting errors in short reads by multiple alignments. Bioinformatics. 2011;27:1455–61. 21471014 10.1093/bioinformatics/btr170

[13] Roberts M, Hayes W, Hunt BR, Mount SM, Yorke JA. Reducing storage requirements for biological sequence comparison. Bioinformatics. 2004;20:3363–9. 15256412 10.1093/bioinformatics/bth408

[14] Williams D, Trimble WL, Shilts M, Meyer F, Ochman H. Rapid quantification of sequence repeats to resolve the size, structure and contents of bacterial genomes. BMC Genomics. 2013;14:537. 23924250 10.1186/1471-2164-14-537 PMC3751351

[15] di Iulio J, Bartha I, Wong EHM, Yu H-C, Lavrenko V, Yang D, et al. The human noncoding genome defined by genetic diversity. Nat Genet. 2018;50:333–7. 29483654 10.1038/s41588-018-0062-7

[16] Georgakopoulos-Soares I, Deng C, Agarwal V, Chan CSY, Zhao J, Inoue F, et al. Transcription factor binding site orientation and order are major drivers of gene regulatory activity. Nat Commun. 2023;14:2333. 37087538 10.1038/s41467-023-37960-5 PMC10122648

[17] Poulsgaard GA, Sørensen SG, Juul RI, Nielsen MM, Pedersen JS. Sequence dependencies and mutation rates of localized mutational processes in cancer. Genome Med. 2023;15. 10.1186/s13073-023-01217-z PMC10436389 37592287

[18] Nordström KJV, Albani MC, James GV, Gutjahr C, Hartwig B, Turck F, et al. Mutation identification by direct comparison of whole-genome sequencing data from mutant and wild-type individuals using k-mers. Nat Biotechnol. 2013;31:325–30. 23475072 10.1038/nbt.2515

[19] Marçais G, Kingsford C. A fast, lock-free approach for efficient parallel counting of occurrences of k-mers. Bioinformatics. 2011;27:764–70. 21217122 10.1093/bioinformatics/btr011 PMC3051319

[20] Rizk G, Lavenier D, Chikhi R. DSK: k-mer counting with very low memory usage. Bioinformatics. 2013;29:652–3. 23325618 10.1093/bioinformatics/btt020

[21] Erbert M, Rechner S, Müller-Hannemann M. Gerbil: a fast and memory-efficient k-mer counter with GPU-support. Algorithms Mol Biol. 2017;12:1–12. 28373894 10.1186/s13015-017-0097-9 PMC5374613

[22] Rhie A, Walenz BP, Koren S, Phillippy AM. Merqury: reference-free quality, completeness, and phasing assessment for genome assemblies. Genome Biol. 2020;21:1–27. 10.1186/s13059-020-02134-9 PMC7488777 32928274

[23] Kokot M, Dlugosz M, Deorowicz S. KMC 3: counting and manipulating k-mer statistics. Bioinformatics. 2017;33:2759–61. 28472236 10.1093/bioinformatics/btx304

[24] Tang D, Li Y, Tan D, Fu J, Tang Y, Lin J, et al. KCOSS: an ultra-fast k-mer counter for assembled genome analysis. Bioinformatics. 2022;38:933–40. 34849595 10.1093/bioinformatics/btab797

[25] Perez G, Barber GP, Benet-Pages A, Casper J, Clawson H, Diekhans M, et al. The UCSC genome browser database: 2025 update. Nucleic Acids Res. 2025;53:D1243–9. 39460617 10.1093/nar/gkae974 PMC11701590

[26] Deorowicz S, Debudaj-Grabysz A, Grabowski S. Disk-based k-mer counting on a PC. BMC Bioinformatics. 2013;14:1–12. 23679007 10.1186/1471-2105-14-160 PMC3680041

[27] Guo K, Gokhale V, Hurley LH, Sun D. Intramolecularly folded G-quadruplex and i-motif structures in the proximal promoter of the vascular endothelial growth factor gene. Nucleic Acids Res. 2008;36. 10.1093/nar/gkn380 PMC2504309 18614607

