# Supplementary material for: MAFcounter: an efficient tool for counting the occurrences of k-mers in MAF files
Source: BMC Bioinformatics. 2025 May 30;26:142. doi: 10.1186/s12859-025-06172-7 (PMC12125892; doi:10.1186/s12859-025-06172-7)
Supplement: Supplementary file 1 — Supplementary Material 1. [file 12859_2025_6172_MOESM1_ESM.docx]

**Supplementary Material**


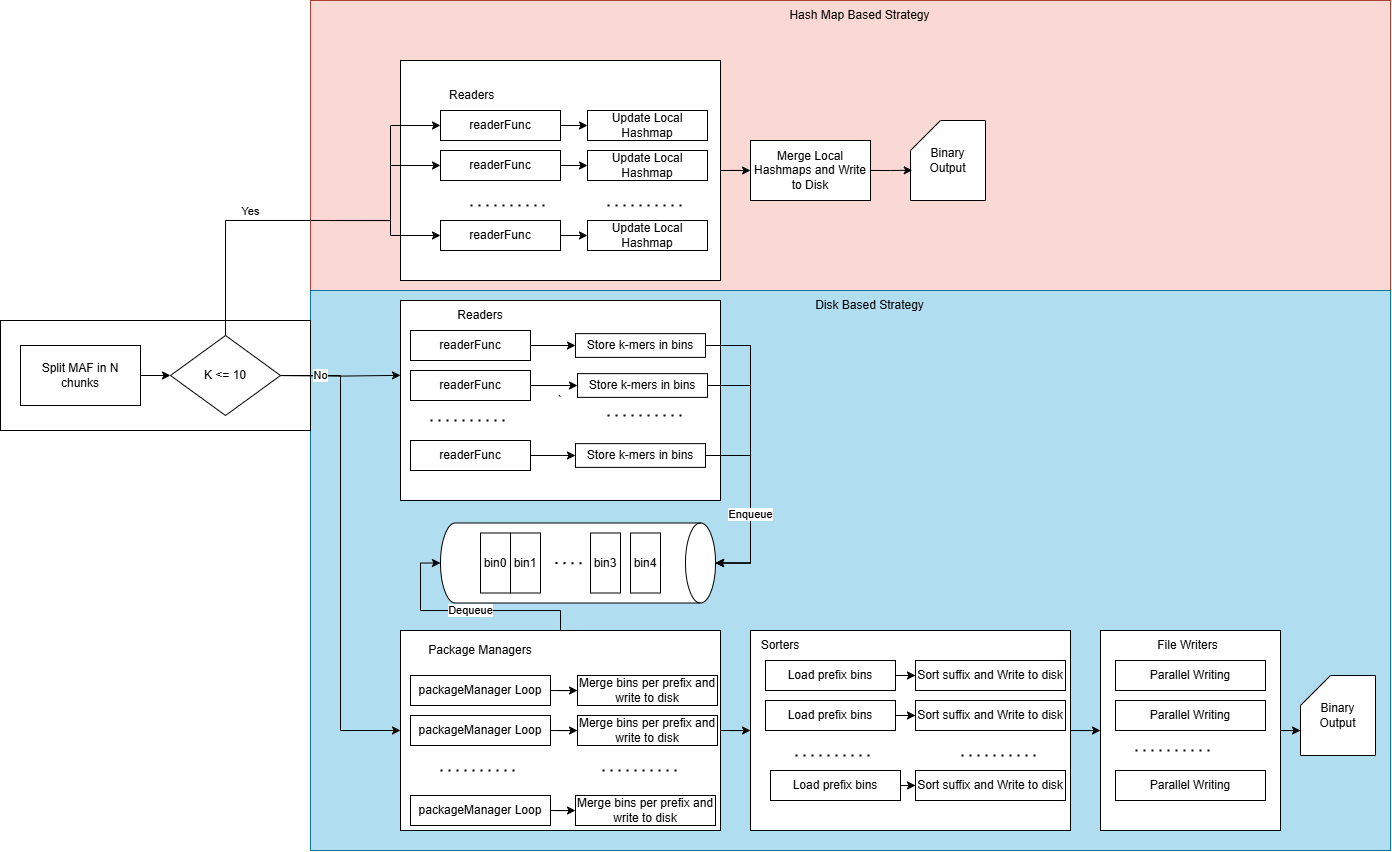


**Supplementary Figure 1: Workflow diagram of MAFcounter illustrating the key steps involved in processing multiple alignment format files for k-mer counting.**


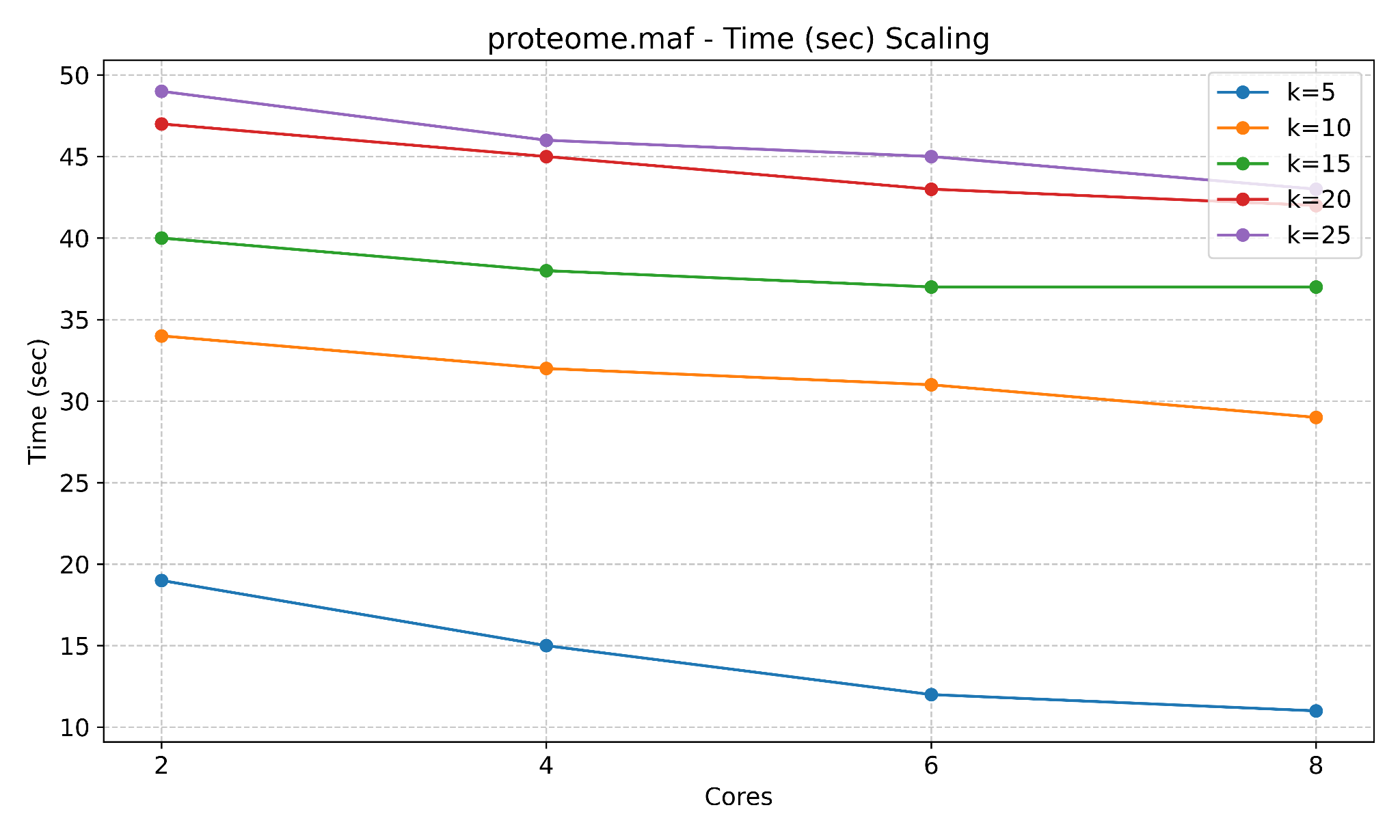


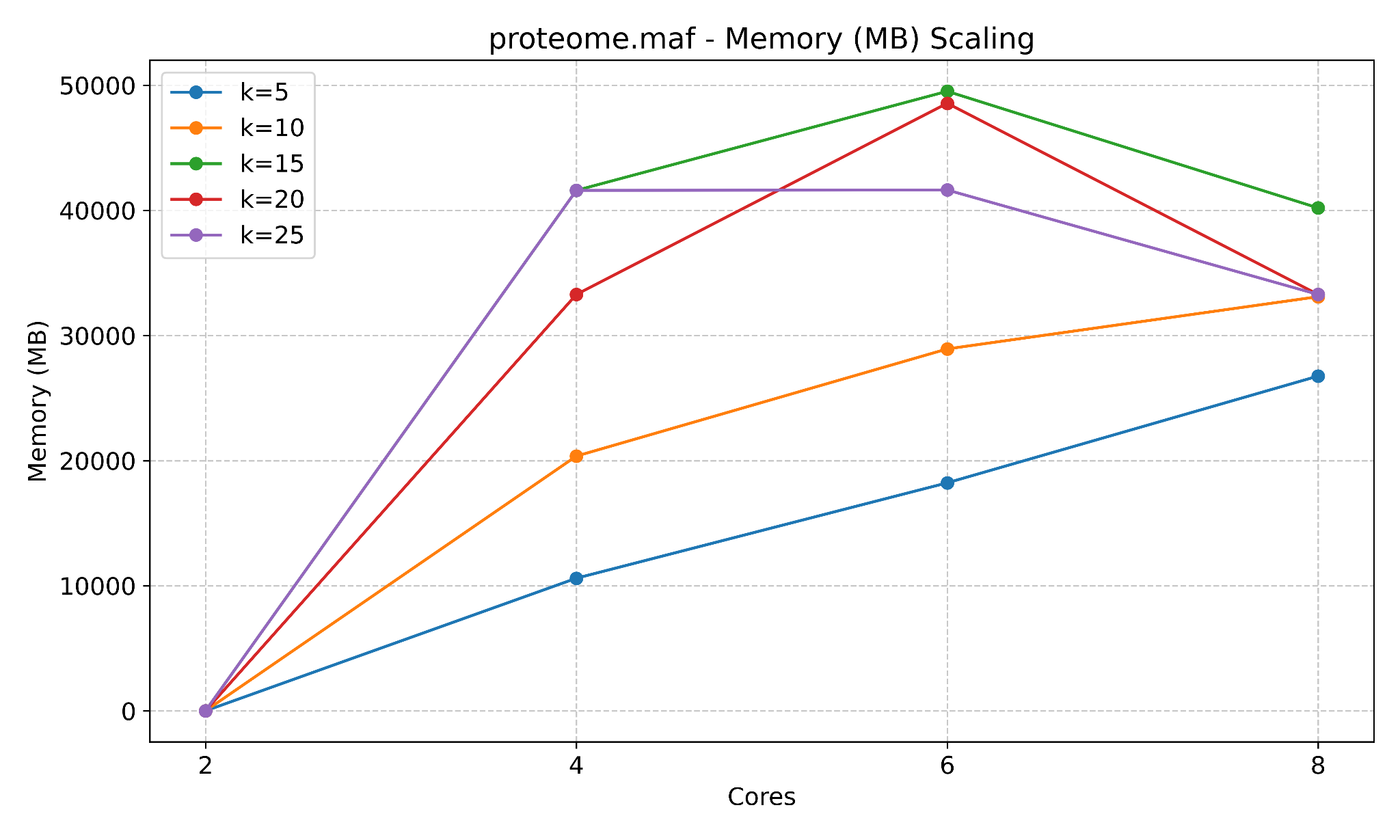


**Supplementary Figure 2: Memory and time performance of MAFcounter on protein MAF files.**


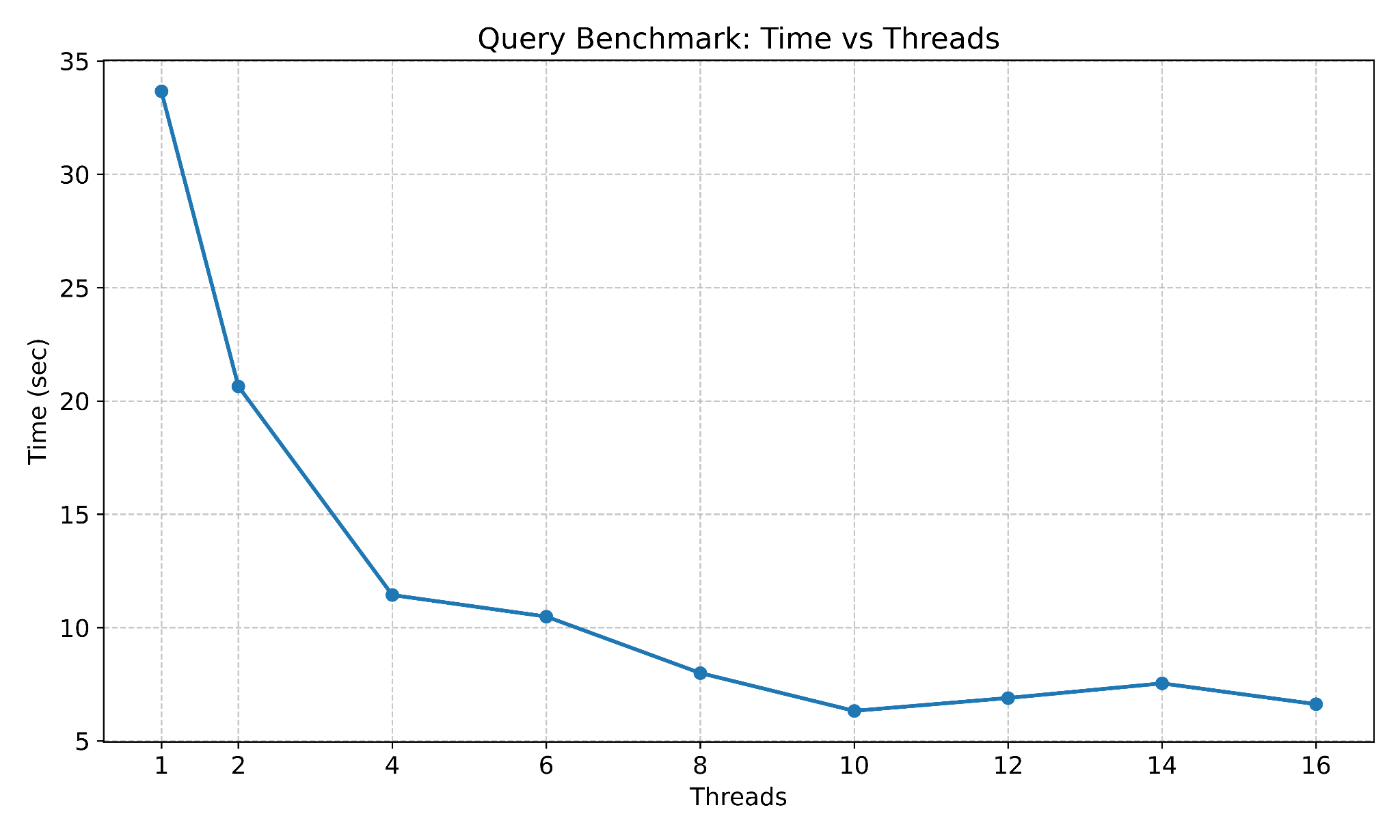


**Supplementary Figure 3: Scaling of execution time for querying feature on 100 k-mers, K=55 on large MAF file using varying threads from 1 , 2 to 16 with step 2.**


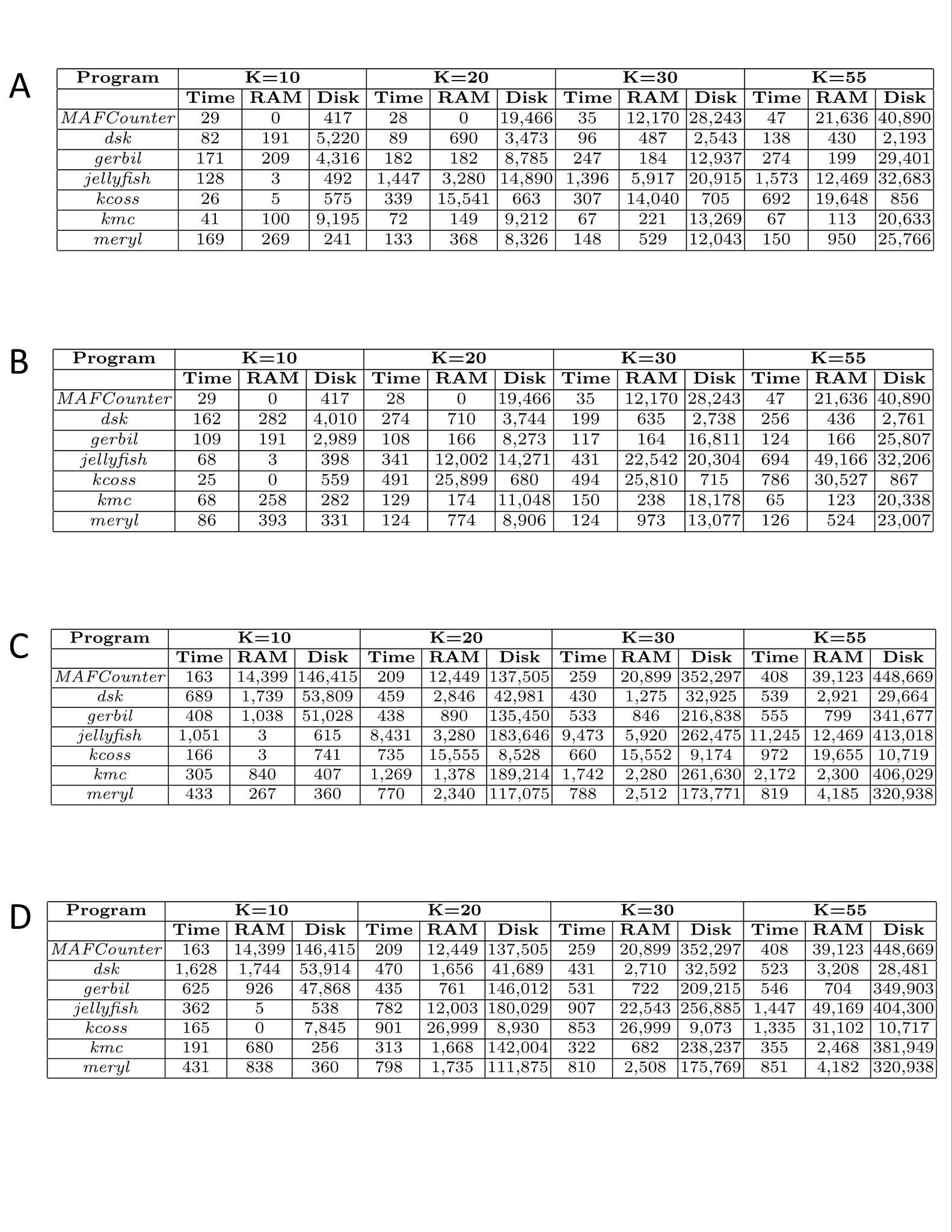


**Supplementary Table 1: Performance comparison against other k-mer counting software. A.** Default parameters on 2GB MAF file. **B.** Fine-tuned parameters on 2GB MAF file. **C.** Default parameters on 25GB MAF file. **D.** Fine-tuned parameters on 25GB MAF file.
